# Volar dislocation of the ulnar head after distal radial fracture: Case report and review of the pertinent literature

**DOI:** 10.1016/j.amsu.2018.10.006

**Published:** 2018-10-10

**Authors:** Nana Nagura, Kiyohito Naito, Ahmed Zemirline, Yoichi Sugiyama, Mayuko Kinoshita, Kenji Goto, Yoshiyuki Iwase, Kazuo Kaneko

**Affiliations:** a Department of Orthopaedics, Juntendo University School of Medicine, 2-1-1 Hongo, Bunkyo-ku, Tokyo, 113-8421, Japan; b Department of Orthopaedic Surgery, Juntendo Tokyo Koto Geriatric Medical Center, 3-3-20 Shinsuna, Koto-ku, Tokyo, 136-0075, Japan; c Hand Center of Brittany, St Grégoire Private Hospital Center, 6 Boulevard de la Boutière, 35760, Saint-Grégoire, France

**Keywords:** Volar dislocation of the ulnar head, Distal radius fracture, Distal radioulnar joint, Triangular fibrocartilage complex, Galeazzi fracture

## Abstract

**Introduction:**

We report the case of volar dislocation of the ulnar head occurred after osteosynthesis for the treatment of distal radius fracture.

**Presentation of case:**

The patient, 68-year-old female, had the dorsal displaced left distal radius fracture and volar dislocation of the ulnar head. Osteosynthesis was performed using a volar locking plate without postoperative immobilization. Two weeks after surgery, volar dislocation of the ulnar head in distal radioulnar joint (DRUJ) was noted on CT. Re-operation, triangular fibrocartilage complex (TFCC) was sutured to the ulnar fovea using a suture anchor, was performed in order to stabilize DRUJ. At 24 months after surgery, left wrist joint pain and the range of motion have improved, and the Mayo wrist score was excellent.

**Discussion:**

Based on the fact that the radius was fractured and the ulna was dislocated in DRUJ at the time of injury, the present case may have been a Galeazzi fracture.

**Conclusion:**

When distal radius fracture is complicated by ulnar instability of DRUJ, active repair of the TFCC function may be necessary to prevent residual postoperative instability.

## Highlights

• We report the case of volar dislocation of the ulnar head occurred after osteosynthesis for a distal radius fracture.
• It is difficult to be diagnosed the DRUJ instability associated with distal radius fracture.
• In this case, active repair of the TFCC function may be necessary to prevent residual postoperative instability.

## 1 Introduction

Triangular fibrocartilage complex (TFCC) injuries, the ulnar notch morphology, and damage of the surrounding soft tissue have been reported as the causes of postoperative distal radioulnar joint (DRUJ) instability associated with distal radius fracture [1], but it is difficult to diagnose these and no consensus has been reached with regard to the selection of conservative or surgical treatment (osteosynthesis of styloid process fracture and repair of TFCC) [2,3].

In cases of DRUJ instability, the frequency of dorsal deviation of the ulnar head is markedly higher, whereas volar dislocation of the ulna is relatively rare [4,5]. We encountered a patient in whom volar dislocation of the ulnar head occurred after osteosynthesis for the treatment of distal radius fracture. We report and discuss the diagnosis and treatment of volar dislocation of the ulnar head in this case.

The work has been reported in line with the SCARE criteria [6].

## 2 Presentation of case

**Fig. 1:**
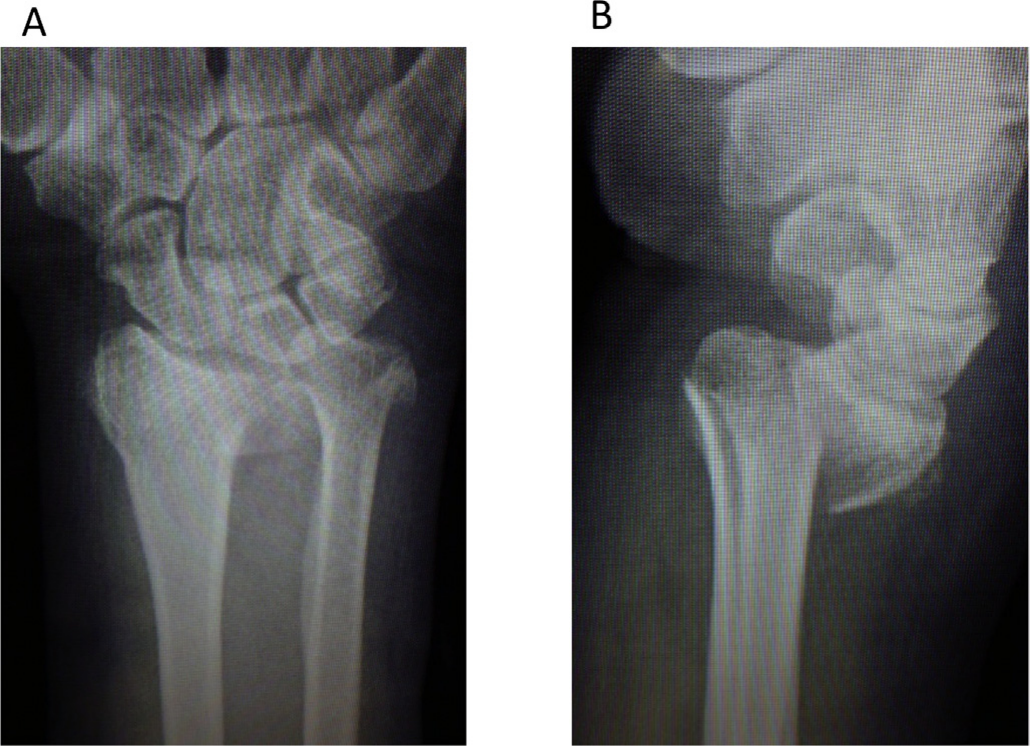
Plain radiography at the time of injury. A: The dorsal dislocated left distal radius fracture associated with ulnar styloid process fracture was observed with a frontal view. B: Volar dislocation of the ulnar head was observed with a lateral view.

**Fig. 2:**
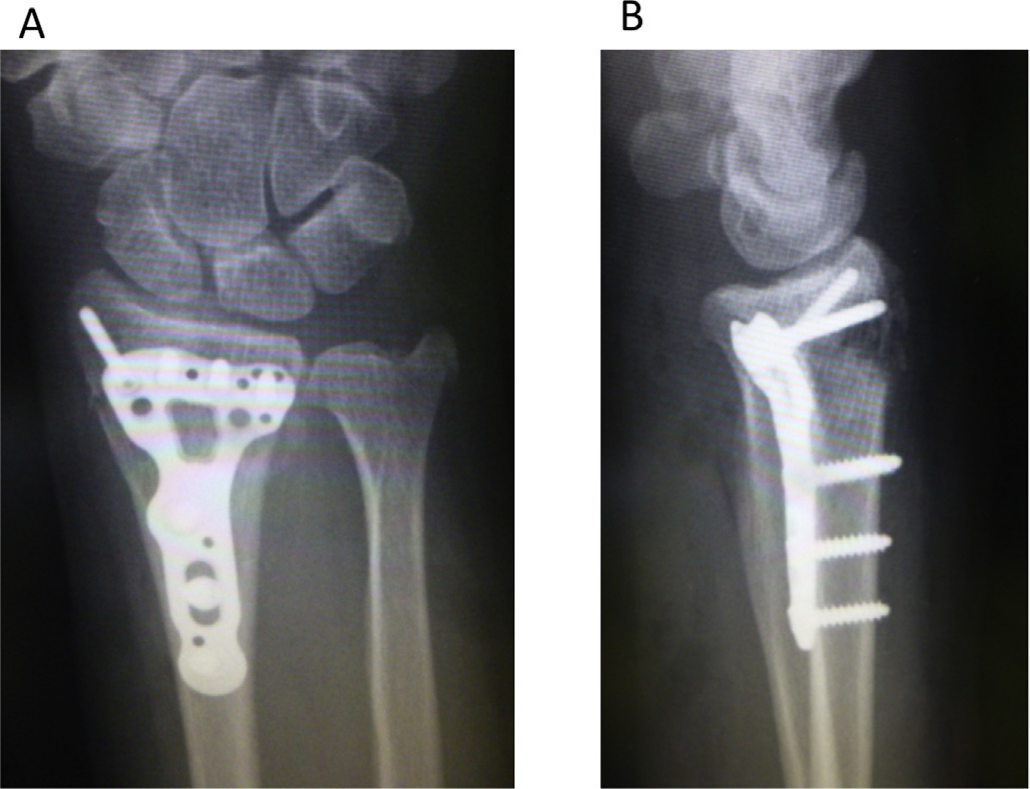
Plain radiography after the first osteosynthesis. Osteosynthesis was applied to the distal radius fracture using a volar locking plate (A: frontal view, B: lateral view).

**Fig. 3:**
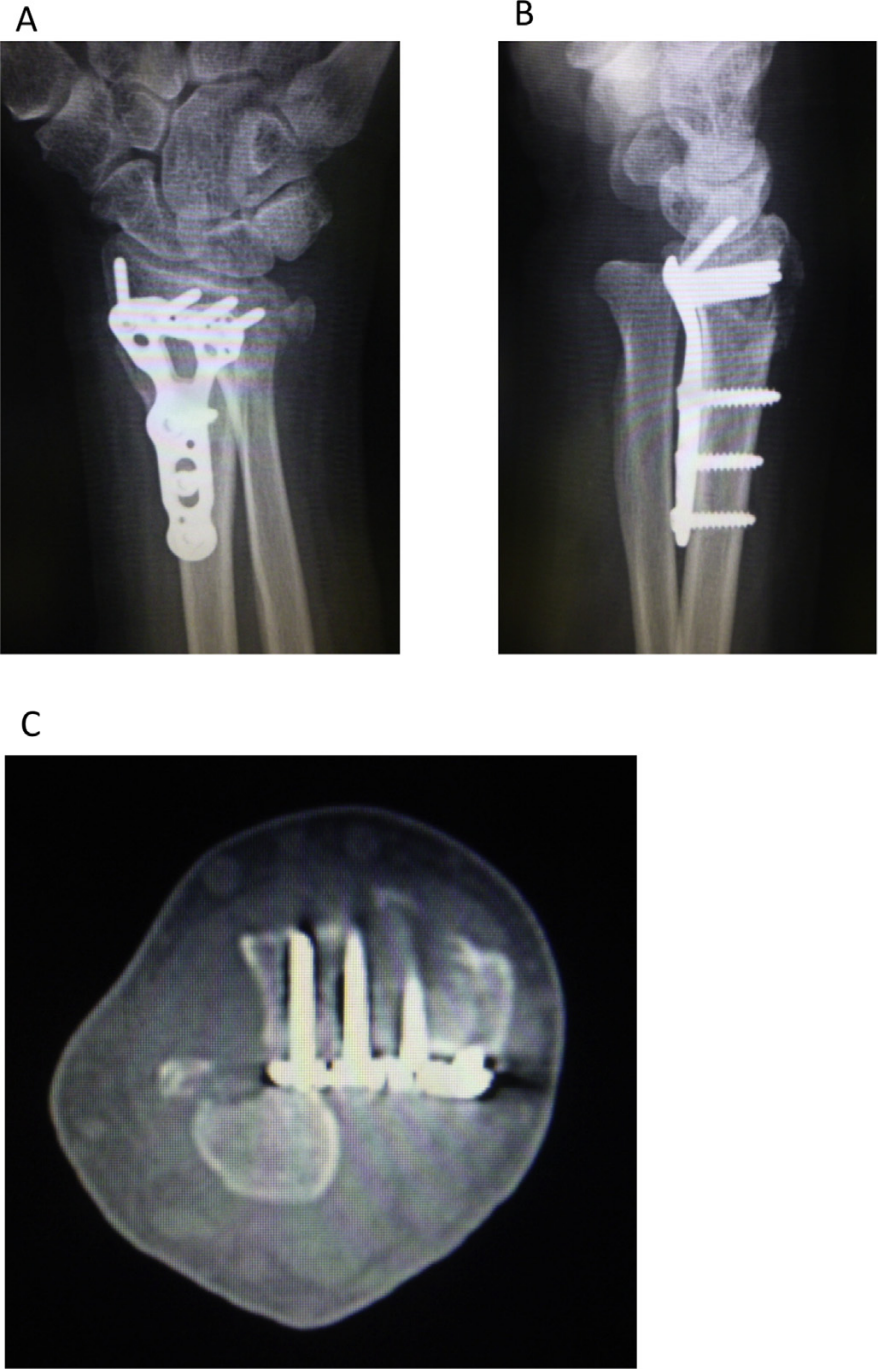
Plain radiography and CT 2 weeks after the first surgery. On plain radiography, incongruency of DRUJ and deviation of the ulnar head were noted (A: frontal view, B: lateral view). With an axial view on CT, volar dislocation of the ulnar head was noted (C).

The left wrist pain developed in a 68-year-old female after falling down the stairs and she visited the emergency service. The dorsal displaced left distal radius fracture associated with ulnar styloid process fracture and volar dislocation of the ulnar head was observed on plain radiography, and the fracture type was A2 according to the AO classification (Fig. 1A and B) [7]. The distal radius fracture was treated with osteosynthesis using a volar locking plate (Aculoc 2, Nihon Medical Next, Osaka, Japan) (Fig. 2A and B). Range of motion exercise was initiated without postoperative immobilization. The left wrist pain developed again at 2 weeks after surgery. Malalignment of the DRUJ was observed on plain radiography (Fig. 3A and B), and volar dislocation of the ulnar head in DRUJ was noted with an axial view on CT (Fig. 3C).

**Fig. 4:**
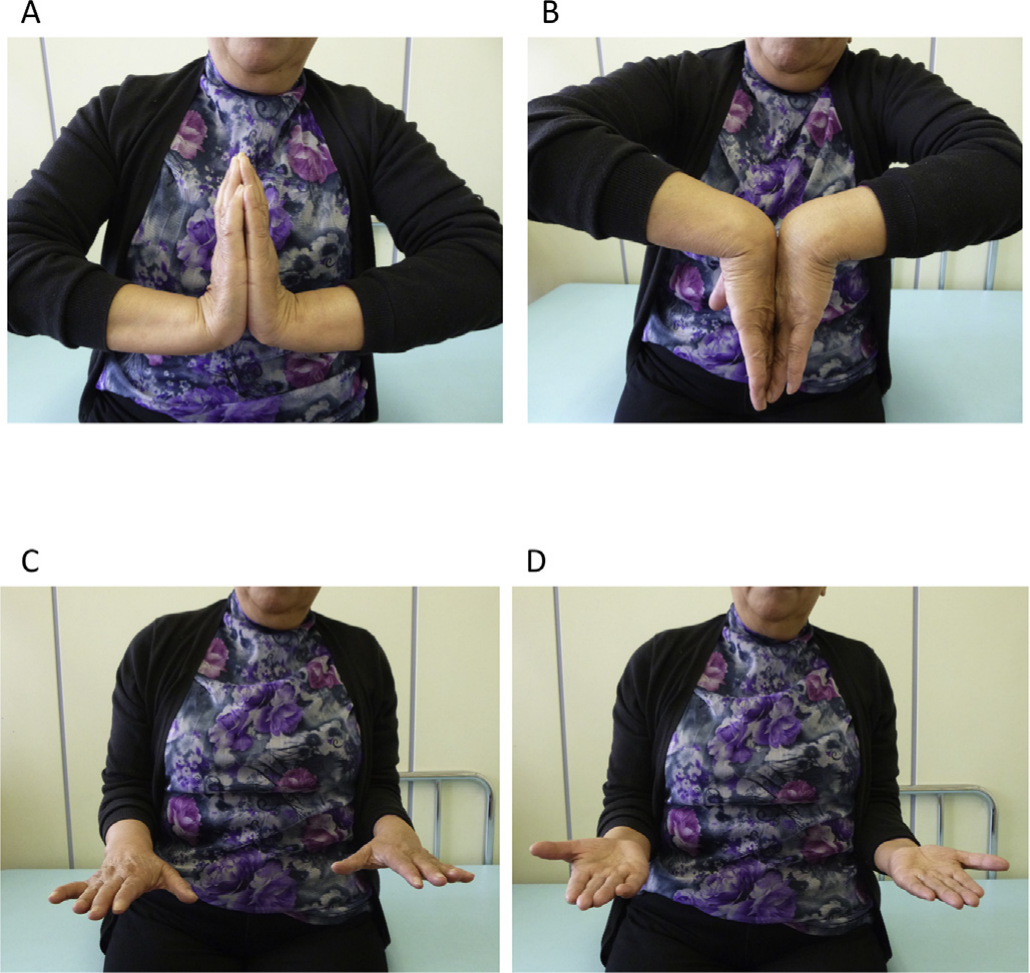
Range of motion of the wrist joint 2 years after surgery. A: Wrist extension. B: Wrist flexion. C: Forearm pronation. D: Forearm supination.

**Fig. 5:**
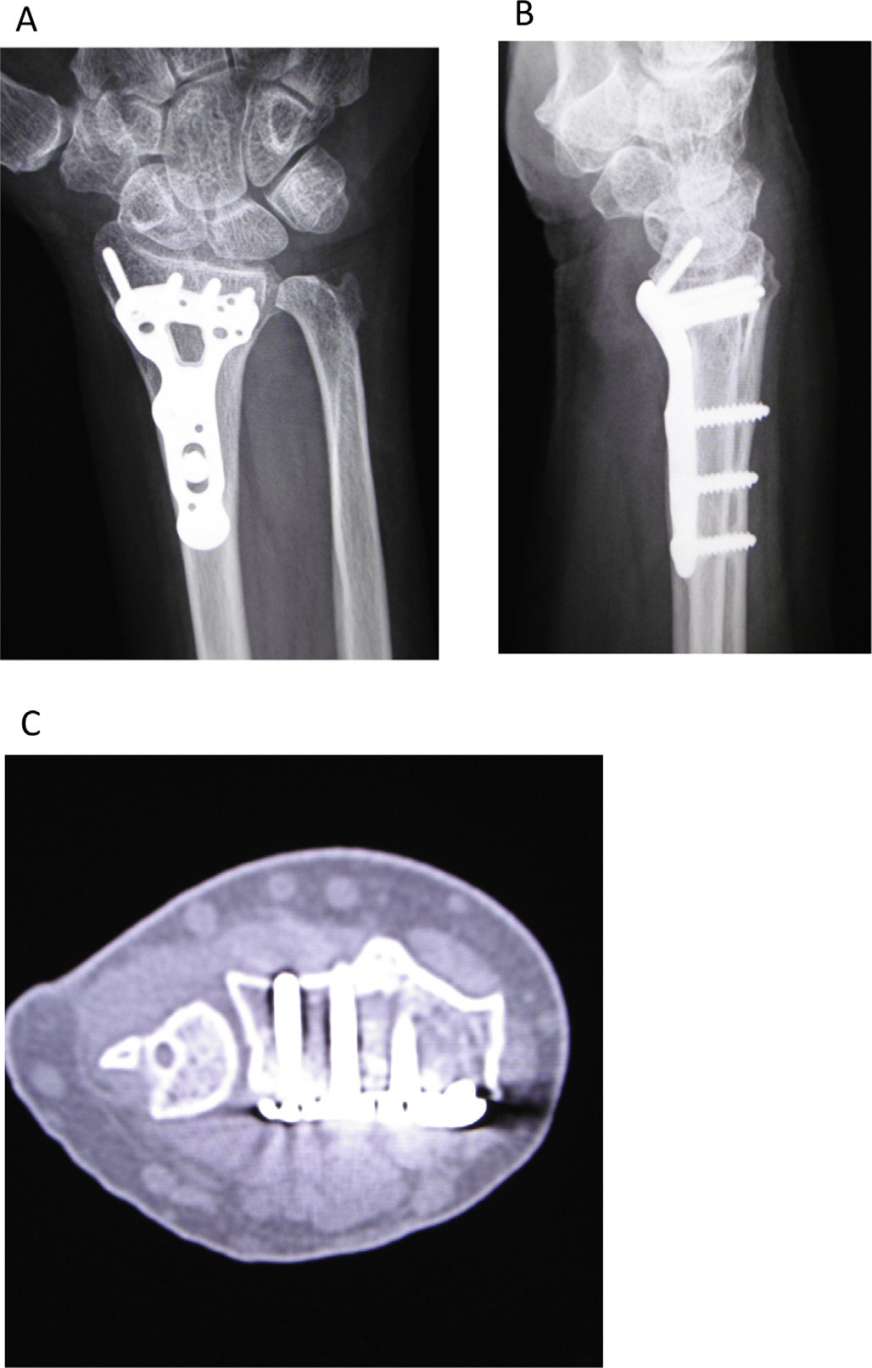
Plain radiography and CT 2 years after surgery. On plain radiography, congruency of DRUJ was favorable (A: frontal view, B: lateral view). With an axial view on CT, the burr hole in the suture anchor insertion site in the ulnar fovea was dilated (C).

Re-operation was performed in order to stabilize DRUJ 3 weeks after osteosynthesis. Rupture of TFCC from the ulnar fovea was observed under direct vision in the wrist joint through the approach between the 5th and 6th compartments from the dorsal side of the wrist joint. TFCC was sutured to the ulnar fovea using a suture anchor (Jugger Knot, 1 mm 3-0, Biomet Japan, Tokyo, Japan), followed by plication of the articular capsule present on the dorsal side. To immobilize postoperative forearm pronation and supination, the ulna and radius were fixed using 1.6-mm Kirschner wire. The Kirschner wire fixing the radioulnar region was removed 4 weeks after surgery, and forearm pronation and supination were permitted. For immobilization, the patient wore an above-elbow splint until 4 weeks after surgery and a below-elbow split until 8 weeks. The range of motion at 2 years after surgery was: wrist extension and flexion, 80°, respectively; forearm pronation and supination, 85°, respectively; grip strength (relative to that on the healthy side), 87.5%; Quick Disabilities of the Arm, Shoulder and Hand (Q-DASH) score 6.82/100; Mayo wrist score, 100/100; showing the return of the condition to that before injury (Fig. 4A–D). Congruency of DRUJ was favorable on plain radiography (Fig. 5A and B) and the burr hole in the suture anchor insertion region in the ulnar fovea was dilated (Fig. 5C).

## 3 Discussion

TFCC contributes to DRUJ stability [8]. Fibers in the superficial and deep layers of the volor/dorsal radioulnar ligament are attached to the styloid process of the ulna and ulnar fovea, respectively [9,10], and the superficial dorsal and deep volar ligaments are retracted by forearm pronation, whereas the superficial volar and deep dorsal ligaments are retracted by supination [5,11]. The position of the ulnar fovea is mostly consistent with the rotation center of DRUJ, and when a strong rotation force is loaded on this region, the deep layer of the radioulnar ligament ruptures and causes DRUJ instability.

Generally, dorsal deviation of the ulnar head is likely to occur when DRUJ instability is noted [5]. Since the deep volar ligament ruptures with forced pronation and causes instability toward the dorsal side, it is considered that the deep volar ligament plays an important role in ulnar instability toward the dorsal side [5]. In contrast, ulnar head instability toward the volar side is not problematic in many cases because not only TFCC but also the distal interosseous membrane (DIOM) plays an important role in the control of this instability [12]. In addition, the distal interosseous membrane extends over the distal radioulnar region and it is located dorsal to the pronator quadratus muscle. The distal oblique bundle (DOB) is attached to the proximal ulna over the dorsal ulnar notch and dorsal wrist articular capsule, being considered the second stabilizer of DRUJ [13]. When tension of the distal interosseous membrane cannot be improved by reduction and fixation of the radius in distal radius fracture or DOB is absent, it is necessary to investigate repair of the injured TFCC, in order to stabilize DRUJ [14,15].

Volar dislocation of the ulnar head was present from the time of injury in this patient, and the instability could not be resolved by treatment of the distal radius fracture with osteosynthesis alone. Actually, rupture of the ulnar fovea over TFCC was observed in the second surgery and DRUJ stability could be achieved by repair of TFCC by suturing it to the ulnar fovea, suggesting that when distal radius fracture is complicated by ulnar dislocation in DRUJ or instability toward the volar side, injury of not only TFCC but also the interosseous membrane should be suspected at the time of injury, and active repair of the TFCC function is necessary to prevent residual postoperative instability.

Based on the fact that the radius was fractured and the ulna was dislocated in DRUJ at the time of injury, the present case may have been a Galeazzi fracture. Generally, Galeazzi fractures have been treated by anatomical reduction of the radius, but recently, DRUJ instability remains in many cases and the necessity of repair of DRUJ instability, such as suture of TFCC, has been reported [16]. Reportedly, DRUJ instability is more likely to remain as the radius fracture site becomes more distal, and the reason for this is as follows: the interosseous membrane is injured only partially in fractures at a 1/3 or more proximal site, but in distal fractures, TFCC and the distal interosseous membrane are continuously injured from DRUJ to the fracture site, and so instability remains [17,18]. When ulnar head deviation (separation between the distal radial bone fragment and ulnar head) complicating distal radius fracture is observed with a lateral view on plain radiography at the time of injury, such as that observed in the present case (Fig. 1B), treatment should be planned on regarding the fracture as a dislocation fracture.

## 4 Conclusion

It is difficult to be diagnosed the DRUJ instability associated with distal radius fracture and no consensus has been reached with regard to the selection of treatment. Based on the fact that the radius was fractured and the ulna was dislocated in DRUJ at the time of injury, the present case may have been a Galeazzi fracture. When distal radius fracture is complicated by ulnar instability of DRUJ, active repair of the TFCC function may be necessary to prevent residual postoperative instability.

## Consent

Written informed consent was obtained from patient for publication of this case report and accompanying images. A copy of the written consents is available for review by Editor-in-Chief of this journal on request.

## Provenance and peer review

Not commissioned, externally peer reviewed.

## Ethical approval

17-250, Ethical Approval of Juntendo University.

## Sources of funding

We have no source.

## Author contribution

All authors have contributed significantly, and that all authors are in agreement with the content of the manuscript.

Nana Nagura, Kiyohito Naito, Mayuko Kinoshita, Kenji Goto, Yoichi Sugiyama and Yoshiyuki Iwase performed operation and ward management; Ahmed Zemirline, Kiyohito Naito and Kazuo Kaneko diagnosed; and Nana Nagura and Kiyohito Naito wrote the paper.

## Conflicts of interest

No funds were received in support of this study.

## Research registry number

Researchregistry3869.

## Guarantor

Dr Kiyohito NAITO.

## Appendix A Supplementary data

checklist checklist
